# Lateral cephalometric radiograph *versus* lateral nasopharyngeal radiograph for quantitative evaluation of nasopharyngeal airway space

**DOI:** 10.1590/2176-9451.19.4.089-093.oar

**Published:** 2014

**Authors:** Suelen Cristina da Costa Pereira, Rejane Targino Soares Beltrão, Guilherme Janson, Daniela Gamba Garib

**Affiliations:** 1 PhD Orthodontic Resident, School of Dentistry — University of São Paulo/ Bauru.; 2 PhD in Orthodontics, School of Dentistry — University of São Paulo/Bauru.; 3 Full professor, Department of Orthodontics, School of Dentistry — University of São Paulo/Bauru.

**Keywords:** Radiology, Nasopharynx, Orthodontics

## Abstract

**Objective:**

This study compared lateral radiographs of the nasopharynx (LN) and lateral cephalometric radiographs (LC) used to assess nasopharyngeal airway space in children.

**Material and Methods:**

One examiner measured the nasopharyngeal space of 15 oral breathing patients aged between 5 and 11 years old by using LN and LC. Both assessments were made twice with a 15-day interval in between. Intergroup comparison was performed with t-tests (P < 0.05).

**Results:**

Comparison between LN and LC measurements showed no significant differences.

**Conclusion:**

Lateral cephalometric radiograph is an acceptable method used to assess nasopharyngeal airway space.

## Abstract (pt)

**Objetivo:**

comparar a radiografia da nasofaringe (RN) e a radiografia cefalométrica lateral (RCL) para avaliação do espaço aéreo nasofaríngeo em crianças.

**Métodos:**

um examinador mediu o espaço da nasofaringe de 15 pacientes respiradores bucais, com idade entre 5 e 11 anos, utilizando a RN e a RCL. Ambas as avaliações foram realizadas duas vezes, com um intervalo de 15 dias. A comparação intergrupos foi realizada por meio do teste *t* (p < 0,05).

**Resultados:**

a comparação entre as medições na RN e na RCL mostrou não haver diferença significante.

**Conclusão:**

Concluiu-se que a radiografia cefalométrica lateral é um método aceitável para avaliar o espaço aéreo da nasofaringe.

## INTRODUCTION

Two modalities of conventional and extraoral radiographs are used to assess nasopharyngeal airway space: lateral nasopharyngeal radiograph (LN), also known as cavum radiography, and lateral cephalometric radiograph (LC). The former is requested most frequently by physicians to assess the nasopharynx of patients with nasal obstruction, whereas the latter has been used for several years in Orthodontics to assess the morphology and development of dental occlusion, including soft and skeletal tissues of the face.^1,2^ Moreover, several authors show that lateral cephalometric radiograph allows one to assess adenoid and dimension of nasopharynx.^1-11^

With the aim of establishing a baseline for measuring nasopharyngeal space on lateral radiographs, McNamara Jr^7^ defined it as the shortest distance between the convex surface of the adenoid (or posterior wall of nasopharynx) and the dorsal surface of the soft palate. Patients with nasopharynx width less or equal to 5 mm reveal apparent airway obstruction. It is used only as an indicator of possible airway impairment. A more accurate diagnosis can be made only by an otorhinolaryngologist during clinical examination.

According to Kohler^12^ and Almeida et al,^3^ lateral cephalometric radiograph and lateral nasopharyngeal radiograph can be used by orthodontists and otorhinolaryngologists as integrated medical-dental examinations. Moreover, they can be obtained during the same procedure, which eliminates the need for additional radiographic exposure.

Ikino et al^2^ assessed the degree of nasopharynx obstruction by means of applying Cohen and Konak^21^ score to both lateral cephalometric radiograph and lateral nasopharyngeal radiograph. His results revealed that similar outcomes were produced by both radiographs in 73.1% of children. The author stated that lateral cephalometric radiograph yields better results in comparison to lateral nasopharyngeal radiograph, since patient's head positioning is standardized in the former. Head position is fixed in LC, which avoids variation in the sagittal and transverse planes and allows a more secure airway analysis without the artifacts produced by head rotation. This information is important since children hardly remain in the desired position. Based on these findings, the authors concluded that LC is the radiograph of choice for assessing nasal obstruction due to equally showing nasopharynx airway and minimizing changes in head positioning.

According to Almeida et al,^3^ computed tomography (CT) is also used in diagnosis of nasopharyngeal obstruction; however, despite being more accurate, it is also more expensive. Montgomery et al^13^ evaluated the results obtained by tomography and concluded that radiographic examination is poor in information. The authors suggest that CT should be used as the gold standard. Conversely, cephalometric radiography should be used to determine whether a more detailed tracking is necessary or not, bearing in mind that this is a two-dimensional and, therefore, limited examination.

No previous study compared lateral nasopharyngeal radiograph with lateral cephalometric radiograph used for quantitative evaluation of nasopharynx. For this reason, the objective of this study was to compare lateral cephalometric radiograph and lateral nasopharyngeal radiograph for a quantitative evaluation of nasopharyngeal airway space.

## MATERIAL AND METHODS

**Figure 1:**
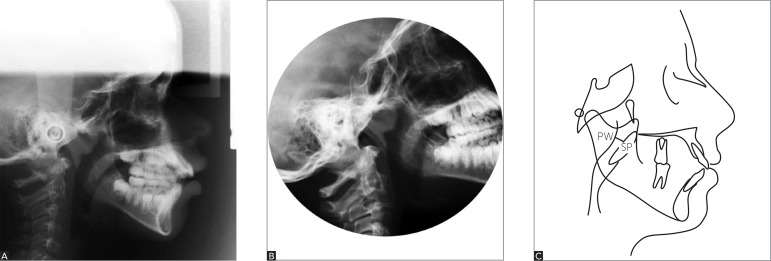
**A**) Nasopharyngeal space measurement expressed in millimeters from the point of the anterior half of the contour of the soft palate (SP) to the nearest point of the pharyngeal wall (PW). **B**) Lateral nasopharyngeal radiograph (LN). **C**) Lateral cephalometric radiograph (LC).

This research was approved by the Federal University of Paraíba (UFPB) Institutional Review Board under protocol 574/06. All research subjects signed an informed consent form. This study assessed the orthodontic records of patients from the School of Dentistry of the University of São Paulo. In selecting the sample, the following inclusion criteria were applied: patients aged between 5 and 11 years old; recent lateral cephalometric radiograph of good quality (Fig 1A); signs of mouth breathing including open mouth posture, short upper lip and everted lower lip; large, varying degrees of narrow face; small nostrils, and poorly developed, deep, narrow palate which demonstrated the need for otorhinolaryngologist analysis.

The final sample comprised 15 patients who were referred to an otorhinolaryngologist for examination of the nasopharynx. A lateral nasopharyngeal radiograph (Fig 1B) was requested for all patients as a supplementary diagnostic tool. The interval between LC and LN was less than three months. Seven patients (46.7%) were males, whereas 8 (53.3%) were females. Patients had a mean age of 8.07 ± 1.58 years (varying from 5 to 11 years) as shown in Table.

Radiographs were manually traced by the same operator using Ultraphan sheets and a 0.35 mm mechanical pencil. Nasopharyngeal space was measured in millimeters with a ruler, from the point of the anterior half of the contour of the soft palate to the nearest point of the nasopharyngeal posterior wall (Fig 1C).

Rotograph Plus (Villa Sistemi Medicali, Buccinasco, Italy) was used for lateral cephalometric radiograph, while Siemens (AG, Munich, Germany) was used for lateral nasopharyngeal radiograph under 10% and 20% image magnification, respectively. Magnification factors were corrected before comparison.

LC was obtained with the patient positioned in a cephalostat with the Frankfurt Horizontal Plane (FHP) parallel to the ground, lips at rest and in centric occlusion. Focus-sagittal midplane distance was 1.52 m and the exposure parameters were 85 KVp, 10 mA and 0.5 to 1 second of exposure time, depending on patient's age.

LN was performed with the child standing in profile with the head horizontally oriented and the mouth closed during inspiration. Focus-sagittal midplane distance was 1.42 m and the exposure parameters were 64 kV and 3.5 mA.

Data were processed in a statistical program (SPSS 11.0) for descriptive and inferential analyses. To calculate error of the method, all radiographs were remeasured after a 15-day interval. The formula proposed by Dahlberg^14^ (Se^2^ = Σd^2^/2n) was used to estimate the order of magnitude of casual errors, while systematic errors were analyzed by paired t-tests, as advocated by Houston.^15^ Independent t-tests were used for intergroup comparison of nasopharynx width in LC and LN (significance level was set at 5%).

## RESULTS

Casual errors were 0.56 and 0.07 for LN and LC, respectively. No statistically significant systematic errors were observed (Table 1).

**Table 1:** Patients' age, analysis of systemic and random errors and comparison between nasopharynx width in LC and LN.

Patients' age (years)						
Mean	Standard deviation		Minimum		Maximum	
8.07	1.58		5		11	
**Analysis of systemic and random errors**						
	**First analysis**		**Second analysis**			
	Mean	SD*	Mean	SD*	P**	Dahlberg
Lateral cephalometric radiograph	11.92	5.41	11.66	5.03	0.212	0.56
Lateral nasopharyngeal radiograph	11.47	3.16	11.49	3.17	0.427	0.07
**Comparison between nasopharynx width in LC and LN**						
Lateral cephalometric radiograph		Lateral nasopharyngeal radiograph				T-test
Mean	SD	Mean		SD		P
11.82	5.22	11.48		3.16		0.385

SD*= Standard deviation

P** = 0.05.

There were no significant differences between nasopharynx measurements in LN and LC (Table 1).

## DISCUSSION

Intragroup analyses showed no significant errors when the first and second measurements for lateral nasopharyngeal radiograph and lateral cephalometric radiograph were compared, thus demonstrating good precision and reproducibility of measurements.

Previous studies reported that nasopharynx evaluation is important to diagnose adenoid size and permeability of airway space.^16,17,18^ This study aimed at comparing nasopharynx width in LC and LN. Correction of image magnification of both types of radiograph allowed comparison of nasopharynx measurements. No significant differences were observed between LC and LN (Table 1), corroborating the study by Ikino et al.^2^ These authors conducted a qualitative analysis of nasopharynx and found an agreement in the degree of airway space obstruction for both types of radiographs, an important factor to consider when dealing with patients who will undergo orthodontic treatment and are likely to have lateral cephalometric radiograph requested for diagnosis, regardless of their respiratory condition. For these patients, evaluation of nasopharynx by means of LC can avoid unnecessary financial and biological costs of taking an extra radiographic exam.^1,19,20^

The technique used to obtain LC yields better results in comparison to LN, since patient's head positioning is standardized in the former. For this reason, it avoids variations in the sagittal, frontal and transverse planes. Rotation of the head may produce undesired effects, especially in children who do not always remain in a desired position.^2^ In addition, LC has the advantage of having a fixed distance between focal point and film.^21^

Major et al^22^ assessed the capability of lateral cephalometric radiographs to diagnose hypertrophied adenoids and obstructed nasopharyngeal airway. They conducted a systematic literature review and concluded that LC performed reasonably well in evaluating adenoid size. Quantitative measures of adenoid area and subjective grading of adenoid size on LCs had reasonable correlations with actual adenoid size.

Barbosa et al^1^ compared the use of LC and endoscopy of nasopharynx to evaluate nasopharynx obstruction. They concluded that LC allows visualization of soft and hard tissue structures , in addition to assessing location, configuration and growth of nasopharynx and adenoid tissue. Moreover, it allows structures closely related to oral cavity and nasopharynx to be visualized. Although this type of radiograph has greater limitations in comparison to two-dimensional interpretation of nasopharynx, it has proved to be effective as a diagnostic tool. This fact was evidenced by the strong correlation between LC results and nasal endoscopy.

## CONCLUSION

No significant differences were found between measurements obtained with lateral nasopharyngeal radiograph and lateral cephalometric radiograph. Lateral cephalometric radiograph proved an acceptable method to evaluate nasopharyngeal airway space by both the orthodontist and the otorhinolaryngologist.
